# Bridge to the future: Important lessons from 20 years of ecosystem observations made by the OzFlux network

**DOI:** 10.1111/gcb.16141

**Published:** 2022-03-22

**Authors:** Jason Beringer, Caitlin E. Moore, Jamie Cleverly, David I. Campbell, Helen Cleugh, Martin G. De Kauwe, Miko U. F. Kirschbaum, Anne Griebel, Sam Grover, Alfredo Huete, Lindsay B. Hutley, Johannes Laubach, Tom Van Niel, Stefan K. Arndt, Alison C. Bennett, Lucas A. Cernusak, Derek Eamus, Cacilia M. Ewenz, Jordan P. Goodrich, Mingkai Jiang, Nina Hinko‐Najera, Peter Isaac, Sanaa Hobeichi, Jürgen Knauer, Georgia R. Koerber, Michael Liddell, Xuanlong Ma, Craig Macfarlane, Ian D. McHugh, Belinda E. Medlyn, Wayne S. Meyer, Alexander J. Norton, Jyoteshna Owens, Andy Pitman, Elise Pendall, Suzanne M. Prober, Ram L. Ray, Natalia Restrepo‐Coupe, Sami W. Rifai, David Rowlings, Louis Schipper, Richard P. Silberstein, Lina Teckentrup, Sally E. Thompson, Anna M. Ukkola, Aaron Wall, Ying‐Ping Wang, Tim J. Wardlaw, William Woodgate

**Affiliations:** ^1^ 2720 School of Agriculture and Environment University of Western Australia Crawley Western Australia Australia; ^2^ Institute for Sustainability, Energy and Environment University of Illinois Urbana‐Champaign Urbana Illinois USA; ^3^ Terrestrial Ecosystem Research Network College of Science and Engineering James Cook University Cairns Queensland Australia; ^4^ College of Science and Engineering James Cook University Cairns Queensland Australia; ^5^ 1994 Faculty of Science University of Technology Sydney Ultimo New South Wales Australia; ^6^ Te Aka Mātuatua – School of Science The University of Waikato Hamilton New Zealand; ^7^ CSIRO Oceans and Atmosphere Canberra Australian Capital Territory Australia; ^8^ School of Biological Sciences University of Bristol Bristol UK; ^9^ ARC Centre of Excellence for Climate Extremes University of New South Wales Sydney New South Wales Australia; ^10^ Climate Change Research Centre University of New South Wales Sydney New South Wales Australia; ^11^ Manaaki Whenua − Landcare Research Palmerston North New Zealand; ^12^ Hawkesbury Institute for the Environment Western Sydney University Penrith New South Wales Australia; ^13^ 5376 Applied Chemistry and Environmental Science RMIT University Melbourne Vic. Australia; ^14^ 1994 Faculty of Science University of Technology Sydney Ultimo New South Wales Australia; ^15^ 10095 College of Engineering, IT & Environment Charles Darwin University Darwin New Territory Australia; ^16^ Manaaki Whenua – Landcare Research Lincoln New Zealand; ^17^ 2220 CSIRO Land and Water Floreat Western Australia Australia; ^18^ School of Ecosystem and Forest Sciences University of Melbourne Richmond Victoria Australia; ^19^ Airborne Research Australia TERN Ecosystem Processes Central Node Parafield South Australia Australia; ^20^ Terrestrial Ecosystem Research Network The University of Queensland Indooroopilly Queensland Australia; ^21^ School of Ecosystem and Forest Sciences University of Melbourne Creswick Victoria Australia; ^22^ Faculty of Sciences University of Adelaide Adelaide South Australia Australia; ^23^ College of Earth and Environmental Sciences Lanzhou University Lanzhou China; ^24^ CSIRO Land and Water Wembley Western Australia Australia; ^25^ Jet Propulsion Laboratory California Institute of Technology Pasadena California USA; ^26^ Centre for Applied Climate Sciences University of Southern Queensland Toowoomba Queensland Australia; ^27^ College of Agriculture and Human Sciences Prairie View A&M University Prairie View Texas USA; ^28^ Department of Ecology and Evolutionary Biology University of Arizona Tucson Arizona USA; ^29^ Queensland University of Technology Brisbane Queensland Australia; ^30^ School of Science Edith Cowan University Joondalup Western Australia Australia; ^31^ Department of Civil, Environmental and Mining Engineering University of Western Australia, Crawley Western Australia Australia; ^32^ Department of Civil and Environmental Engineering University of California Berkeley California USA; ^33^ CSIRO Oceans and Atmosphere Aspendale Victoria Australia; ^34^ ARC Centre for Forest Values University of Tasmania Hobart Tasmania Australia; ^35^ School of Earth and Environmental Sciences The University of Queensland Brisbane Queensland Australia; ^36^ CSIRO Space and Astronomy Kensington Western Australia Australia

**Keywords:** agroecosystem, disturbance, eddy covariance, flux network, global change, modelling, remote sensing, stress, TERN

## Abstract

In 2020, the Australian and New Zealand flux research and monitoring network, OzFlux, celebrated its 20^th^ anniversary by reflecting on the lessons learned through two decades of ecosystem studies on global change biology. OzFlux is a network not only for ecosystem researchers, but also for those ‘next users’ of the knowledge, information and data that such networks provide. Here, we focus on eight lessons across topics of climate change and variability, disturbance and resilience, drought and heat stress and synergies with remote sensing and modelling. In distilling the key lessons learned, we also identify where further research is needed to fill knowledge gaps and improve the utility and relevance of the outputs from OzFlux. Extreme climate variability across Australia and New Zealand (droughts and flooding rains) provides a natural laboratory for a global understanding of ecosystems in this time of accelerating climate change. As evidence of worsening global fire risk emerges, the natural ability of these ecosystems to recover from disturbances, such as fire and cyclones, provides lessons on adaptation and resilience to disturbance. Drought and heatwaves are common occurrences across large parts of the region and can tip an ecosystem's carbon budget from a net CO_2_ sink to a net CO_2_ source. Despite such responses to stress, ecosystems at OzFlux sites show their resilience to climate variability by rapidly pivoting back to a strong carbon sink upon the return of favourable conditions. Located in under‐represented areas, OzFlux data have the potential for reducing uncertainties in global remote sensing products, and these data provide several opportunities to develop new theories and improve our ecosystem models. The accumulated impacts of these lessons over the last 20 years highlights the value of long‐term flux observations for natural and managed systems. A future vision for OzFlux includes ongoing and newly developed synergies with ecophysiologists, ecologists, geologists, remote sensors and modellers.

## Graphical abstract

The Australian and New Zealand ecosystem observation network—OzFlux—celebrated 20 years making ecosystem measurements in 2020. Important insights and new knowledge from this 20‐year period were identified and discussed as eight key lessons. Our vision for the future of OzFlux is also discussed, and includes training the next generation and providing ecosystem data to improve understanding of Australia's and New Zealand's rapidly changing environments.

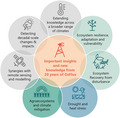

## 1 INTRODUCTION

Ecosystem flux networks are demonstrating their increased relevance to society's most significant sustainability challenges, particularly those linked to global change (Baldocchi, 2019; Long, 2020). The need for better information and knowledge about energy, water and carbon budgets in natural and managed ecosystems, and the underlying processes that govern these budgets, is growing as the world looks to land‐based carbon sequestration to help achieve net zero greenhouse gas emissions. Quality data and expert knowledge will be critical to building confidence in these options for managing net emissions in a changing climate.

OzFlux, the regional flux monitoring network covering Australia and New Zealand, began in the late 1990s in anticipation of these global challenges, especially climate change (see next section for more detail). Two decades on from its establishment in 2001, OzFlux has matured into a network that supports research about Australia's and New Zealand's unique ecosystems, provides key data for Southern Hemisphere terrestrial systems, and observations for some ecosystems subject to an extreme and highly variable climate. The OzFlux community has created an observing network and platform to enable scientific discoveries by generations of researchers and to deliver relevant and robust data and information for researchers, resource managers and policymakers, now and into the future. Through OzFlux, this research community has also transformed its approach to data sharing, acknowledging the challenges this can involve and developing solutions to address these, alongside demonstrating the significant benefits that flow from ensuring that data complies with FAIR (Findable Accessible Interoperable Reusable) principles (Wilkinson et al., 2016). OzFlux provides an example to other flux networks and research communities of the importance of data sharing.

The combined research infrastructure of OzFlux and similar regional networks around the world (Mizoguchi et al., 2009; Novick et al., 2018; Park et al., 2018; Rebmann et al., 2018) contribute to the globally coordinated FLUXNET network (Baldocchi et al., 2001). Like OzFlux, this global network of micrometeorological ‘flux towers’ that use the eddy covariance method, provide observations to advance the understanding and simulation of processes across the past, present and future for a wide array of the world's ecosystems. These continuous, long‐term and standardised measurements are critical for detecting ecosystem stress, recovery from disturbance, and resilience to climate change, as well as exploring the causes and effects of longer‐term climate trends and interannual variability—a goal unattainable with short‐term records (Baldocchi et al., 2018). In‐situ flux tower and remote sensing observations are being combined to upscale from site to regional and global scales (e.g. Cleugh et al., 2007; Jung et al., 2020; Schimel & Schneider, 2019), contributing valuable data‐driven diagnoses of how climate change affects terrestrial carbon and water cycles (e.g. Piao et al., 2020). Similarly, combining in situ flux tower measurements, manipulation experiments and satellite remote sensing are advancing knowledge of how climate extremes affect the carbon cycle (Sippel et al., 2018). See Chapin et al. (2006) for definitions of carbon cycle terms used in this paper. FLUXNET’s global database of ecosystem‐scale observations are being used to evaluate and improve the processes represented in many ecophysiological, hydrological and land surface models (LSMs), improving the regional and global Earth System models used around the world (e.g. Ziehn et al., 2020).

Vegetation of Australian and New Zealand ecosystems have evolved in geographic isolation, geological stability, long‐term aridity and fire‐prone environments. In Australia, these conditions have resulted in a unique flora with scleromorphic properties enabling existence in arid climates on old, highly weathered, low‐nutrient soils and frequent fire (Fox, 1999). As a result, endemism in Australian flowering plants and gymnosperms is extremely high at 93% and 96% relative to global floras (Chapman, 2009). The Australian climate envelope differs from that of Europe, most of North America, Asia and South America, being, on average, warmer and drier (both in terms of rainfall and vapour pressure deficit; VPD) but also subject to larger interannual variations in rainfall and VPD than experienced across much of the globe. While much of Australia is arid or semi‐arid, there are also regions that experience extremely large annual rainfall totals. The associated rainforests are also extensive in the tropical north‐east. Unlike other continents, Australian vegetation is dominated by sclerophyllous, evergreen, woody species—species that are poorly represented in classifications of global plant functional types. Multiple interactions between these factors of low soil nutrient content, extreme interannual variability in rainfall, temperature and VPD across most of Australia, and systemic differences in vegetation attributes (for example, wood density, SLA, photosynthetic nitrogen‐use efficiency—see Table 1) result in divergences of relationships among climate variables, carbon and water fluxes, resource‐use efficiencies (for example Radiation Use Efficiency; Ponce‐Campos et al., 2013) and vegetation attributes across the continents. Of the nine key ecophysiological attributes listed in Table 1, eight are statistically different from typical values of European, North American and global vegetation. Such reasoning underpins the rationale for, and importance of, the OzFlux network.

**TABLE 1:** A comparison of selected vegetation traits across Australian, North American and European plant species, and a combined data set (Global)

Trait	Australia	North America	Europe	Global	Trans
Wood density (g cm^−3^)	0.69 ± 0.0069 (890) a	0.63 ± 0.011 (317) b	0.55 ± 0.019 (46) c	0.67 ± 0.0054 (1253)	
Sapwood specific hydraulic conductivity (kg s^−1^ m^−1^ MPa^−1^)	0.54 ± 0.11 (90) a	0.45 ± 0.11 (65) a	−0.53 ± 0.21 (23) b	0.37 ± 0.077 (178)	ln
Specific leaf area (m^2^ kg^−1^)	1.61 ± 0.033 (386) a	2.68 ± 0.034 (407) b	2.75 ± 0.027 (394) b	2.36 ± 0.024 (1187)	ln
Foliar N (mg g DW^−1^)	12.40 ± 0.38 (330) a	21.39 ± 0.51 (330) b	21.54 ± 0.57 (253) b	18.18 ± 0.31 (913)	
*V*_cmax_ mass basis (nmol CO_2_ g^−1^ s^−1^)	−1.10 ± 0.039 (165) a	−0.49 ± 0.068 (55) b	−0.75 ± 0.13 (24) b	−0.93 ± 0.037 (244)	ln
Stomatal conductance (mmol m^−2^ s^−1^)	4.98 ± 0.053 (192) a	5.49 ± 0.057 (173) b	5.41 ± 0.19 (21) b	5.23 ± 0.040 (386)	ln
*A*_max_ Maximum assimilation rate (mass basis) (nmol CO_2_ g^−1^ s^−1^)	4.16 ± 0.042 (192) a	4.75 ± 0.045 (176) b	5.16 ± 0.13 (40) c	4.51 ± 0.035 (408)	ln
*A*_sat_/N (=photosynthetic nitrogen use efficiency; PNUE)	5.21 ± 0.17 (192) a	6.41 ± 0.20 (170) b	8.15 ± 0.60 (40) c	6.01 ± 0.14 (402)	
Foliar ^13^C discrimination	22.00 ± 0.27 (63) a	20.30 ± 0.17 (141) b	20.15 ± 0.21 (33) b	20.70 ± 0.13 (237)	

Data retrieved from multiple publicly available data sets, but especially the TRY plant trait data set (Max Planck Institute for Biogeochemistry) and GLOPNET (Macquarie University) and the Diefendorf et al., global carbon discrimination data base. Means followed by a different letter within a row are significantly different from each other. Numbers of replicates shown in parentheses. Data which have been transformed are noted in the ‘Trans’ column. Unpublished analyses of data by D. Eamus and B. Murray.

The aim of this paper is to describe the unique and most important insights, and new knowledge contributed by the OzFlux network over its 20‐years of operation. Through a series of short ‘lessons’, we show how Australian and New Zealand ecosystems and landscapes interact with land management practices, climate variability and climate change, with a focus on the following: (1) ecosystem response, resistance and resilience to disturbance and stress; (2) ecosystem processes that modulate water availability, runoff and productivity and (3) net greenhouse gas emissions and the potential for these ecosystems to mitigate climate change and support ecosystem services and food production in the future. This aim reflects that our primary audience for these lessons is the ecosystem research community, however we anticipate that those ‘next users’ of the knowledge, information and data that networks such as OzFlux support may also find benefit from these insights. In distilling the key lessons learned, we also identify where further research is needed to fill knowledge gaps and improve the utility and relevance of the outputs from OzFlux.

## 2 THE GENESIS OF OzFlux

The OzFlux journey began in the early 1990s when Australian and New Zealand researchers embarked on longer‐term micrometeorological field campaigns and studies in agricultural, natural and modified forest, native grassland and wetland ecosystems. This research revealed gaps in our knowledge of ecosystem dynamics and feedbacks with climate and hydrology at multiple timescales, across the diverse landscapes of New Zealand and Australia (Campbell & Williamson, 1997; Cleugh et al., 2007; Hollinger et al., 1994; Leuning et al., 2004). Through long‐term international collaborations, Australian and New Zealand researchers learned from the scientific advances of similar research programs developing overseas, which themselves benefitted from the history of pioneering micrometeorological research in Australia and New Zealand. This included major contributions to the theory and methods for making eddy covariance (i.e. flux) measurements, data processing and analysis, all of which were necessary for enabling long‐term, autonomous flux monitoring (Finnigan et al., 2003; Leuning et al., 1982; Webb et al., 1980). High quality, in situ measurements of ecosystem fluxes and stores of water, carbon and nutrients were also being sought to calibrate and validate remotely sensed observations in these unique landscapes and ecosystems. Flux data were also being incorporated into biophysically realistic LSMs, such as the CABLE LSM within Australia's global climate and Earth system model (Australian Community Climate and Earth System Simulator, Ziehn et al., 2020).

**FIGURE 1:**
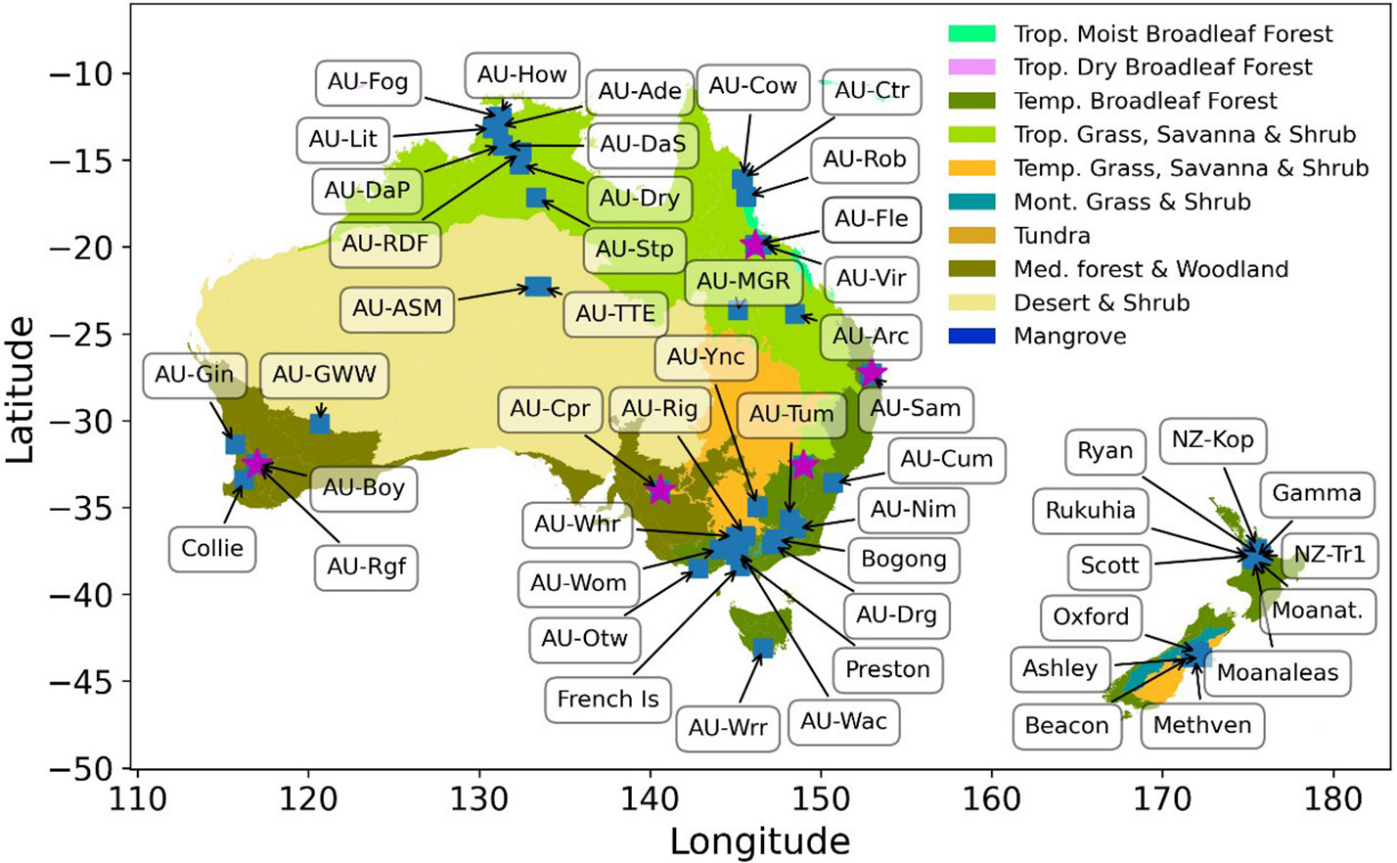
OzFlux tower sites labelled with Fluxnet ID where available (blue square) and critical zone observatories (purple star) across Australia and New Zealand, including major biome types defined using the ‘Ecoregions2017’ data set from Dinerstein et al. (2017) licensed under CC‐BY 4.0. For a current list of active sites and their specifications visit www.ozflux.org

The need for continuous ecosystem data led to the first establishment of flux towers in several ecosystems around Australia (Figure 1): (1) a managed wet temperate forest in south‐eastern Australia (Tumbarumba, Bago State Forest, New South Wales); (2) a semi‐arid subtropical savanna site in western Queensland (Virginia Park, Leuning et al., 2005); (3) a wet temperate forest in southeast Australia (Wallaby Creek in Victoria, Kilinc et al., 2012); (4) a tropical savanna woodland of the Northern Territory (Howard Springs, Eamus et al., 2001) and (5) a high‐rainfall, tropical rainforest in Far North Queensland (Cape Tribulation). In New Zealand, the focus was on understanding the impacts of land management and hydro‐climatic factors on ecosystem (especially soil) carbon stock changes (Hunt et al., 2004; Mudge et al., 2011; Nieveen et al., 2005), with longer‐term tower sites established at both agricultural (Hunt et al., 2016; Rutledge et al., 2017) and wetland (Goodrich et al., 2017) sites (Mudge et al., 2011; Nieveen et al., 2005; Owen et al., 2007).

These foundational flux tower sites sowed the seeds of OzFlux, which expanded to a continental network when TERN (Terrestrial Ecosystem Research Network) was funded in 2009. This funding provided the capital and institutional investment needed to support the ‘hard’ infrastructure of around a dozen flux towers and supersites across Australia (Beringer et al., 2016; Karan et al., 2016). Equally important, it provided the dedicated and sustained support for ‘soft’ infrastructure needs such as training for early career researchers; the data management infrastructure to comply with FAIR data principles (Wilkinson et al., 2016); data curation and data processing to ensure consistency across the network; data quality control and assurance; and data discoverability and data access (Beringer et al., 2017; Isaac et al., 2017).

**FIGURE 2:**
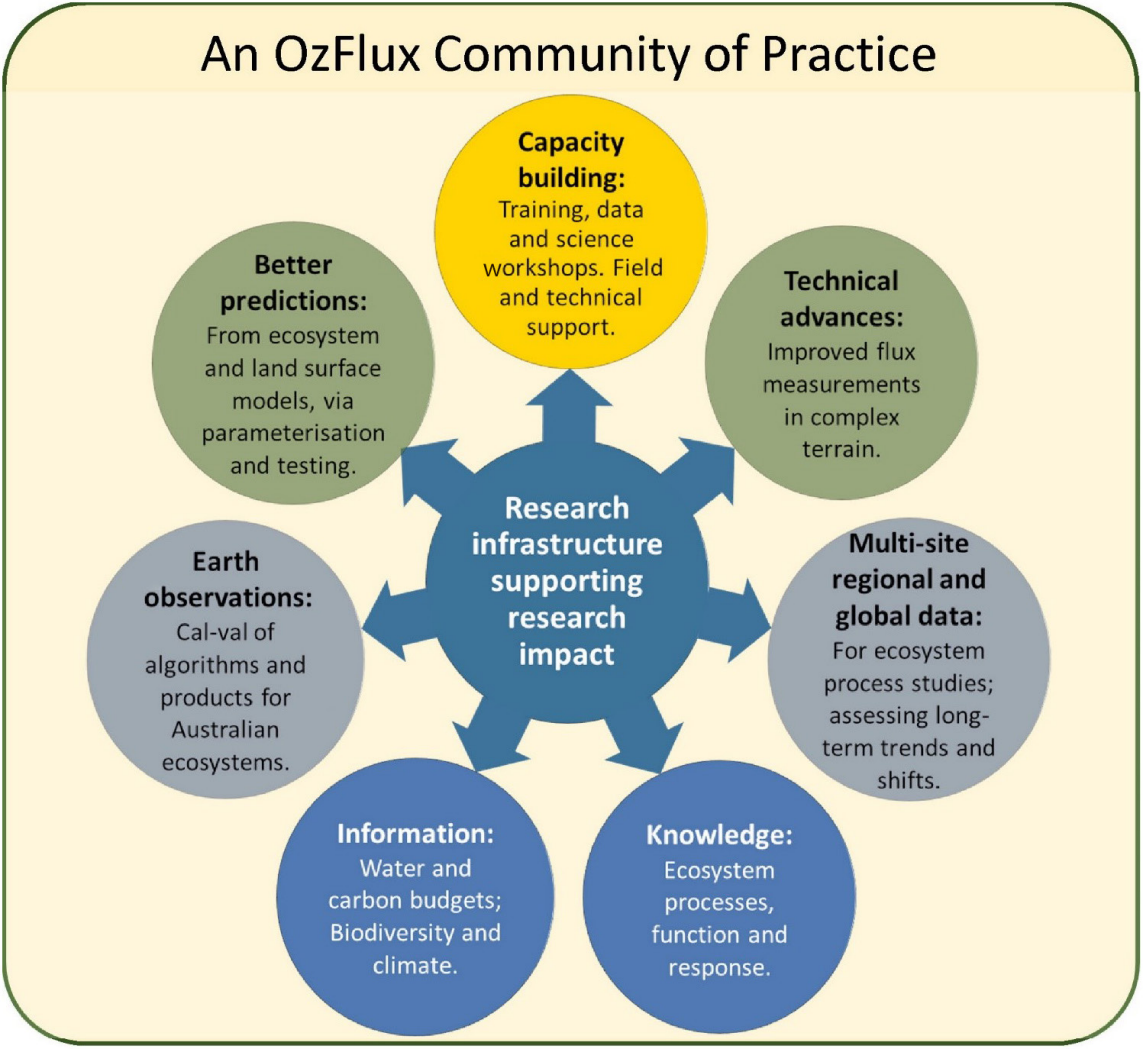
Summary of the significant scientific and technical outcomes from the OzFlux network after two decades: Blue relates to discovery, information and knowledge outcomes; grey outcomes relate to assessments across site, regional and global scales; yellow refers to the capacity building outcomes for researchers and green indicates technical outcomes for observations and modelling

With the addition of new flux towers in ca. 2010 and the development of integrated data processing systems (Isaac et al., 2017), OzFlux has run as a truly regional network since 2010. Historically, Australian OzFlux researchers have largely focussed on natural and forested ecosystems, whereas New Zealand OzFlux research has concentrated on greenhouse gas budgets and emissions from agricultural systems, including drained peatlands. The long‐term investment in OzFlux has led to significant and diverse research outcomes and impacts as summarised in Figure 2. The following sections explore some of the key lessons and outcomes from OzFlux in more detail, and how they have contributed to global understanding in their respective scientific space.

## 3 LESSON 1—OzFlux ECOSYSTEMS EXTEND OUR UNDERSTANDING OF THE CLIMATE SPACE

**FIGURE 3:**
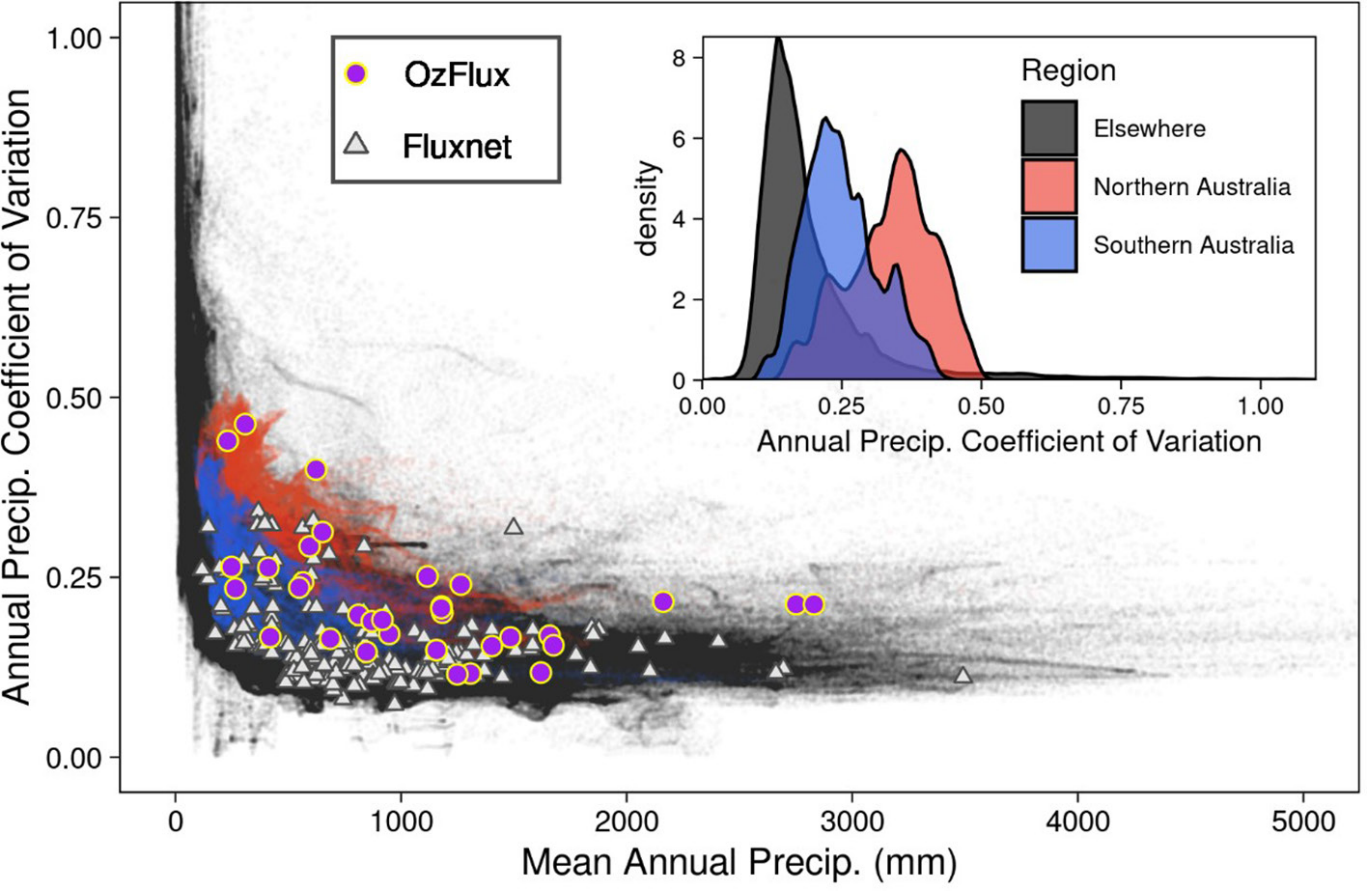
The coefficient of variation of annual precipitation plotted against mean annual precipitation (global gridded data) for the period 1981–2010 with probability distributions showing Northern Australia, Southern Australia, rest of the world (inset). Precipitation data were extracted from the TerraClimate dataset (Abatzoglou et al., 2018) at 0.09° resolution for regions between 60°S and 80°N. For visualisation regions where mean annual precipitation was less than 5 mm yr^−1^ are removed. Northern (red) and Southern Australia (blue) are differentiated by the 28°S Latitude parallel. The corresponding climates of FluxNet (grey triangle) and OzFlux sites (purple circles) are shown

Terrestrial ecosystems measured in OzFlux span a vast bioclimatic space from alpine to tropical, coastal to central desert. OzFlux sites include some of the hottest sites within FLUXNET, while also covering a rainfall range from 260 to 3930 mm yr^−1^ on average (Beringer et al., 2016), ranging from water‐ to energy‐limited sites (De Kauwe et al., 2019; van der Horst et al., 2019). Many sites are subject to high temperatures, including frequent heatwaves, and high interannual variability in rainfall. In fact, both the Northern and Southern Australian regions have distributions of mean annual precipitation (MAP) variability that are much higher than the rest of the world (Figure 3), and OzFlux sites measure across a very large range of MAP and in areas with higher MAP co‐efficient of variation not captured by FLUXNET sites (Figure 3). Moreover, OzFlux includes sites with a very large spatial range in VPD, greater than 6 kPa (Renchon et al., 2018), allowing exploration of vegetation responses to high VPD that goes well beyond the conditions currently experienced by most ecosystems in the Northern Hemisphere (Grossiord et al., 2020). It is sometimes argued that Australian and New Zealand vegetation and its management is unique, with the implication that it is difficult to use data from these ecosystems to inform our understanding of vegetation function on other continents (see also Table 1). However, in this time of accelerating climate change, the network becomes a natural laboratory to develop inform a global understanding of vegetation responses to increasingly extreme climate conditions, including to high temperatures not yet experienced in most parts of the world (Hutley et al., 2011; van der Horst et al., 2019).

Australia's and New Zealand's climate can vary greatly from one year to the next due to hemispheric‐scale modes of variability (e.g. El Niño Southern Oscillation, Southern Annular Mode, Indian Ocean Dipole; Rogers & Beringer, 2017) and the influence of regional weather phenomena (e.g. Tropical Cyclones, East Coast Lows or West Coast Troughs; Beringer & Tapper, 2000) with important impacts on the continent's terrestrial carbon balance (Teckentrup et al., 2021)—as illustrated for precipitation in Figure 3. Regional and continental weather events can trigger pronounced variations in rainfall distribution that result in large seasonal and interannual variations of leaf area index (LAI), gross primary productivity (GPP) and ecosystem respiration (ER) (Cleverly, Eamus, Luo, et al., 2016; Cleverly et al., 2019; Griebel et al., 2017; Haverd, Ahlström, et al., 2016; Haverd, Smith, Trudinger, et al., 2016; Hinko‐Najera et al., 2017; Li et al., 2017; Renchon et al., 2018; Xie et al., 2019). They also result in seasonal fluctuations between mild and wet maritime winds and hot and dry continental winds from the Australian mainland. These shifts not only affect plant productivity, but also provide methodological challenges for comparing annual budgets that have been constructed from flux tower observations (Griebel et al., 2016; Griebel, Metzen, Pendall, et al., 2020).

Recent heatwaves during a prolonged drought across southern Australia have proven valuable to examine the individual and compounded effects of extreme temperature and water stress on the hourly and daily exchange of CO_2_ and H_2_O in temperate forests and woodlands. A synthesis across seven OzFlux sites during the record‐breaking heatwave in the ‘Angry Summer’ of 2012/2013 demonstrated that temperate woodlands became net sources of CO_2_ on a daily average during the most intense part of the heatwave. This response was attributed to increased ER during hotter days and nights and to a reduction in the magnitude and number of hours of carbon uptake (van Gorsel et al., 2016). However, large reductions (up to 60%) in GPP were only observed in water‐limited woodlands, while forests with access to deep soil water were able to sustain photosynthesis near to or beyond baseline levels at the cost of increased water loss through evapotranspiration (Griebel, Bennett, et al., 2020; van Gorsel et al., 2016). These results highlight that the potential for temperate forests and woodlands to remain net carbon sinks will not only depend on the responses of photosynthesis to warmer temperatures, but also on soil water availability and on the concomitant responses of ER.

High temperatures and associated deficits in atmospheric vapour pressure provide challenges for the ability of plants to regulate water loss and to maintain photosynthesis. A synthesis across 17 OzFlux wooded ecosystems demonstrates strong alignment between the thermal optima of GPP and mean daytime air temperatures, indicating ecosystem scale photosynthesis has adjusted to past thermal regimes (Bennett et al., 2021). Although it currently seems that GPP in Australian broadleaf evergreen forests is buffered against small increases in air temperature, the shape of this relationship and the response of ER to rising temperatures will determine the sustainability of Australian carbon sinks into the future (Bennett et al., 2021; Duffy et al., 2021; Griebel, Bennett, et al., 2020; van Gorsel et al., 2016).

The cooling effect of transpiration protects leaves from heat damage during extreme temperatures, and decoupling of photosynthesis from transpiration has been demonstrated in experimental manipulations of young eucalypt trees (Drake et al., 2018). However, a meta‐analysis across OzFlux sites highlighted that the confounding role of increasing VPD on transpiration had blurred any conclusive evidence of decoupling between photosynthesis and transpiration at the ecosystem scale (De Kauwe et al., 2019).

Whether transpiration continues or is suppressed during heatwaves is crucial for coupled land‐atmosphere processes and impacts on regional climate. If vegetation can sustain transpiration during heatwaves, a negative feedback results in a cooling and moistening of the atmospheric boundary layer. Conversely if transpiration ceases, the resulting positive feedback leads to heating and drying of the boundary later and amplifies the heatwave regionally. Understanding these mechanisms is therefore critical in understanding how climate change will be expressed as heatwaves over vegetated surfaces. It also means that models representing the impact of global climate change regionally, and on terrestrial ecosystems, must represent these processes and mechanisms.

## 4 LESSON 2 – ECOSYSTEM RECOVERY FROM DISTURBANCE

Disturbances in Australia and New Zealand can include fire, cyclones and severe storms, pests, disease, agricultural management and land‐use change, all of which have varying levels of impact on ecosystem carbon cycling. Baldocchi (2008) discussed how the ratio of GPP to ER (i.e. GPP/ER) of disturbed sites is lower than that of undisturbed sites. When plotting GPP and ER from OzFlux sites, Beringer et al. (2016) showed that only a few had a low GPP/ER ratio, despite several sites in the network with a history of disturbance. While much of the network was established in undisturbed sites, many have been subject to natural or managed disturbance over the past 20 years. The apparent resilience of these ecosystems to disturbance is an important aspect of their longer‐term carbon balance in response to global change, which is discussed further in lesson 4.

Bushfire is one of the most widespread causes of ecosystem disturbance across Australia, having shaped adaptations in vegetation across the continent for over 80 million years, similar to southern Africa and in contrast to the more recent development of fire in the Mediterranean region and the Americas (Carpenter et al., 2015; Cleverly et al., 2019). In tropical Northern Australian mesic savannas, bushfires are frequent, with 30% of the total savanna land area burned annually (Beringer et al., 2011, 2015). This fire regime directly affects carbon emissions and productivity due to canopy loss (Beringer et al., 2007). Global climate change is expected to further increase extreme fire weather, and thus greenhouse gas emissions, which will further reduce the savanna carbon sink (Beringer et al., 2003; Duvert et al., 2020). By contrast, land management, which reduces fire frequency and intensity (e.g. by shifting fires from the late to the early dry season) is reducing greenhouse gas emissions at landscape scales in the tropical savanna (Edwards et al., 2021). Fire in Australia's tropical savannas has been shown to reduce the strength of the monsoon, and hence affect regional climate, by modifying the dynamics of the atmospheric boundary layer via changes in the partitioning of the surface energy budget (Beringer et al., 2003, 2015; Gorgen et al., 2006; Lynch et al., 2007; Richards et al., 2011; Wendt et al., 2007). Clearly, lessons learned about vegetation‐climate‐fire relations in the Australian tropical savanna are highly relevant for understanding global change (Lehmann et al., 2014) and are applicable to fire‐prone ecosystems in the United States, southern Europe and Africa.

Where fires in northern Australia are frequent and of low intensity, fire in southern Australia tends to be infrequent and very destructive (Cleverly et al., 2019). Fire in temperate and Mediterranean‐type ecosystems of southern Australia turns them initially into a CO_2_ source, with source strength depending on vegetation and climate (Sun et al., 2017; Wardlaw, 2021). This was illustrated by recent estimates that the bushfires burning in Australia between November 2019 and January 2020 emitted 715 million tonnes (range 517–867) of CO_2_ into the atmosphere (about twice Australia's annual net anthropogenic CO_2_ emissions; van der Velde et al., 2021). Fire in a tall eucalypt forest in southwest Tasmania switched the ecosystem to a net CO_2_ source for the first year post‐fire, despite the survival of canopy trees and prolific seedling regeneration (Wardlaw, 2021). In mallee ecosystems of South Australia, which consist of several species of multi‐stemmed *Eucalyptus*, it can take over 3 years post‐fire before net ecosystem productivity (NEP = GPP‐ER) recovers to pre‐fire levels, despite fires having little effect on respiration or nutrient cycling (Sun et al., 2015, 2020). By contrast, NEP in mesic tropical savanna ecosystems of northern Australia returns to pre‐fire status in 3–4 months post‐fire (Beringer et al., 2007). The knowledge provided from this research into bushfires in Australia, including regional differences between the northern and southern parts of the continent, is important for understanding how these ecosystems adapt to changing climates. It is particularly useful for determining whether they remain carbon sinks in the long‐term as fire frequency and intensity changes, and for informing and improving Earth system models, many of which are poor at simulating fire.

**FIGURE 4:**
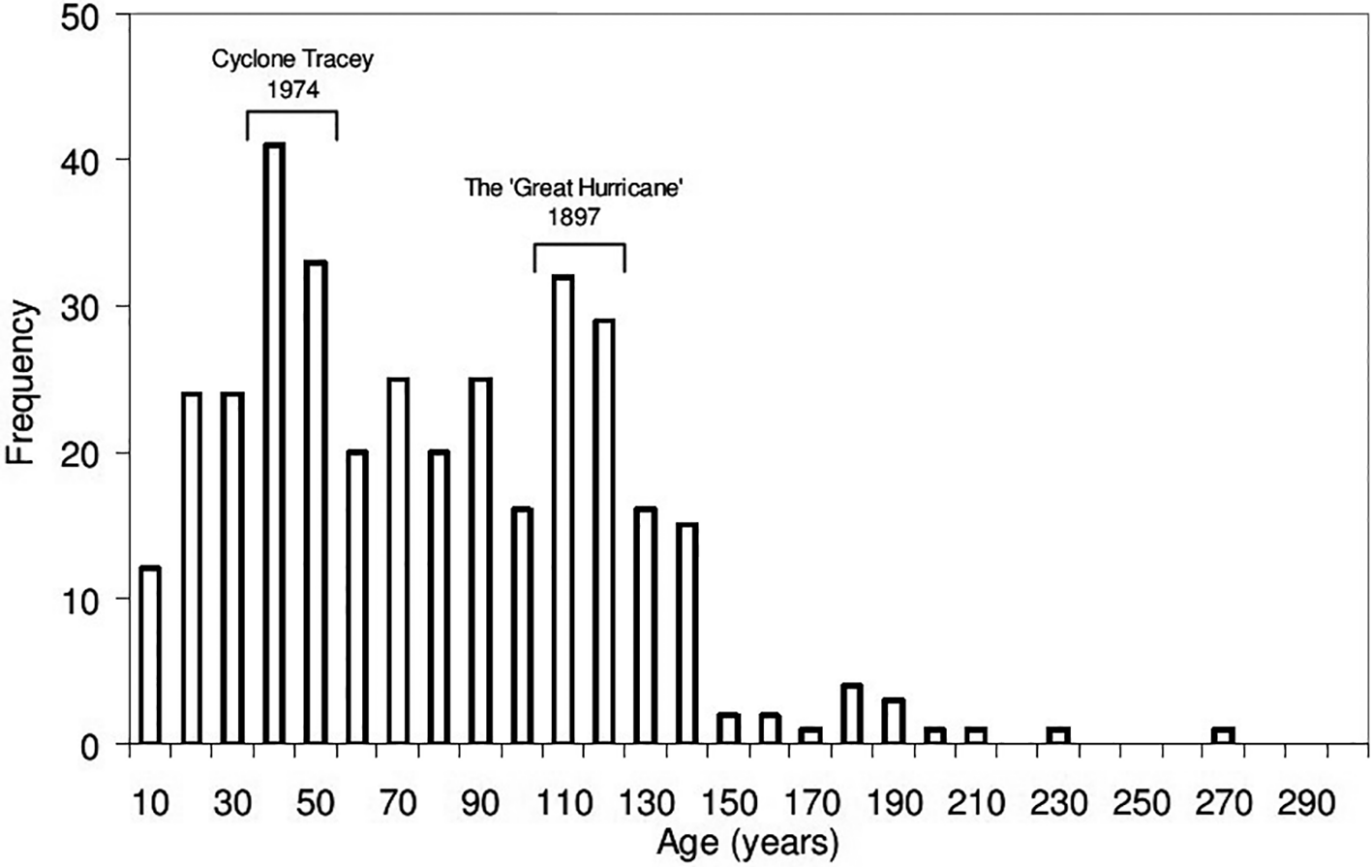
Frequency distribution of the age of Eucalyptus and Corymbia trees at the Howard Springs flux site (number of trees) for trees >2 cm DBH (diameter at breast height) showing history of disturbance at the site. A relationship between age and tree size has been established for these ecosystems (Prior et al., 2004) and was used to convert DBH to age. Figure reproduced with permission from Hutley and Beringer (2011)

Tropical cyclones largely affect OzFlux sites in northern Australia and occur infrequently, but when they do, they often cause great destruction. For example, Cyclone Monica in April 2006 affected 10,400 km^2^ of savanna across northern Australia, resulting in mortality and severe structural damage to 140 million trees (Cook & Nicholls, 2009; Hutley et al., 2013). The current tree‐stand structure at the long‐term savanna flux site at Howard Springs is likely to have been affected by previous cyclones as shown by the age distribution of tree diameter (Figure 4) (Hutley & Beringer, 2011; O’Grady et al., 2000). Recruitment and stand regrowth post‐1974 are likely to explain the high NEP typically measured at the site (2–4 Mg C ha^−1^ y^−1^) (Beringer et al., 2016; Duvert et al., 2020; Eamus et al., 2001), which is indicative of this site's continued state of disequilibrium and underscores the importance of understanding site history for interpreting NEP. The likely impacts of increased storm intensity include larger recruitment pulses, thus larger episodic CO_2_ emissions, potentially with a smaller sequestration potential of these ecosystems.

Whereas the effects of fire and cyclones have been well characterised in some sites across the OzFlux network, gaps remain in our knowledge about the consequences of changing fire intensity and regimes on ecosystem carbon and water budgets more broadly across New Zealand and Australia. There is the added challenge that some very intense fires can destroy the very infrastructure that measures the effects of fire on these fluxes, further limiting our understanding. Gaps also exist in our understanding of the impacts on ecosystems of very infrequent cyclones, particularly in the tropical rainforests of Far North Queensland. Additionally, few or no OzFlux measurements have provided a detailed carbon budget for disturbance by pests, disease, or land‐use change. These knowledge gaps can be difficult to fill because many but not all disturbances require the serendipity of being in the right place at the right time. This reinforces the need for continuous measurements over many decades, to increase the chances of being in the right place at the right time.

## 5 LESSON 3—THE EFFECT OF DROUGHT AND HEAT STRESS ON ECOSYSTEM CARBON AND WATER BALANCES

The primary stress events in natural and managed ecosystems across Australia and New Zealand are related to water availability, usually in the form of short‐ or long‐term meteorological drought, and many ecosystems have adapted to withstand prolonged episodes of water limitation. The last 20 years has seen significant increases in temperature (the Australian continent has warmed by 1.44 ± 0.24°C since 1910) and a resultant increase in more frequent and intense heatwaves (Australian Bureau of Meteorology & CSIRO, 2020). A shift towards drier conditions across Australia's southern regions, especially in the April to October ‘cool season’, has been shown to be the most sustained large‐scale change since the late 19th century and are linked to the effects of anthropogenic climate change on the circulation systems that affect Australia's seasonal weather patterns. Lower rainfall, combined with warming and increased evaporative demand are exacerbating the reductions in water availability in rivers and in the soil (Australian Bureau of Meteorology & CSIRO, 2020). The drier conditions observed in southeast and southwest Australia over the last two decades have contributed to regional patterns of warming with a positive feedback effect on increased evaporative demand. Therefore, flux monitoring in Australia and New Zealand has been critically placed to capture the response of native and managed ecosystems to the occurrence of these emerging trends in interannual and more frequent stress events (Cleverly, Eamus, Luo, et al., 2016; Moore et al., 2018) (see lessons 1, 4 and 8).

The impact of drought has been particularly evident in semi‐arid Australia, where ecosystems have shifted from weak CO_2_ sinks into CO_2_ sources (Ma et al., 2016; Qiu et al., 2020). The pivot point at which an ecosystem switches from a CO_2_ sink to a CO_2_ source can depend on the vegetation properties; for example, the *Acacia spp*. dominated woodland near Alice Springs, in the arid centre of Australia, remain a net CO_2_ sink as long as the annual rainfall exceeds 260 mm (site average is 300 mm yr^−1^), whereas the nearby hummock grasslands become a CO_2_ source if the annual rainfall falls below the pivot point of 506 mm yr^−1^ (Tarin, Nolan, Eamus, et al., 2020).

Ecosystems can also respond to drought stress by regulating their water use via phenotypic plasticity as observed in *Eucalyptus obliqua* at the Wombat State Forest in south‐eastern Australia, where leaf water potential at the turgor loss point was lowered through osmotic adjustment during a short‐term summer drought (Pritzkow et al., 2020). Other drought response mechanisms include partial drought deciduousness, where LAI is reduced to minimise the surface area for water loss, which also increases the Huber value (ratio of sapwood area to leaf area) during extended drought (Meyer et al., 2015; Pritzkow et al., 2020). Individual species may also behave differently when subject to similar stresses, as shown at Cumberland Plain, where the melaleuca stand maintained higher canopy conductance and transpiration under VPD and moisture stress than the neighbouring eucalypt stand (Griebel, Metzen, Boer, et al., 2020).

Drought events in New Zealand, although less intense than those typically experienced in Australia, can still reduce ecosystem carbon uptake. For example, a short‐term meteorological drought turned an intensively grazed dairy pasture into a net CO_2_ source (Kim & Kirschbaum, 2015; Kirschbaum et al., 2015; Rutledge et al., 2015). The intensive grazing that characterises these systems regularly removes pasture dry matter. Pasture regrowth and carbon uptake via photosynthesis following grazing is limited during drought conditions, leading to net carbon loss (Kirschbaum et al., 2017; Wall et al., 2019). In contrast to highly managed agroecosystems, native peatland bogs in New Zealand's Waikato region are able to maintain a strong carbon sink even during drought (Goodrich et al., 2017) likely due to ample soil moisture stores.

**FIGURE 5:**
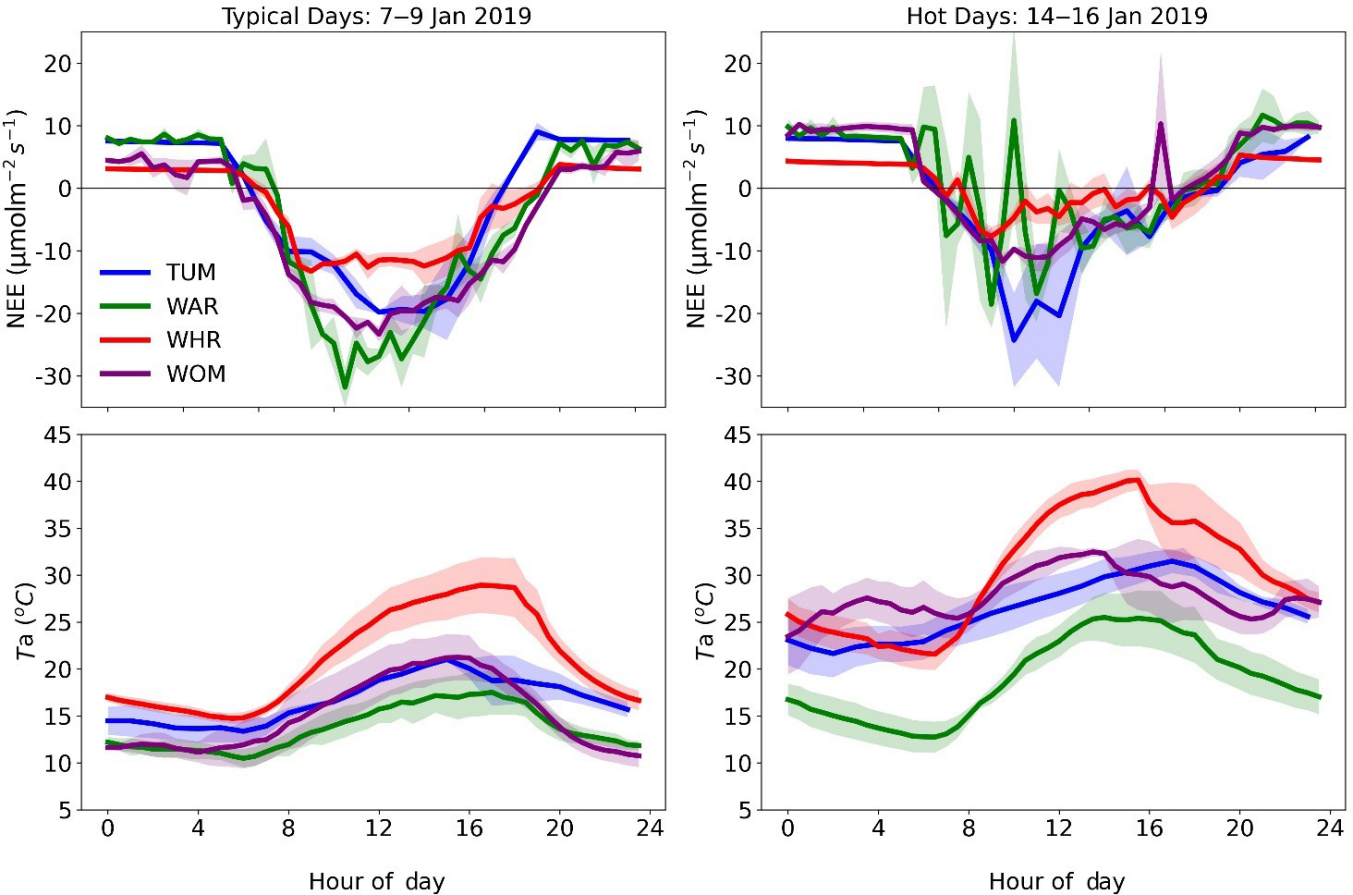
Diurnal average (±standard error) net ecosystem exchange (NEE) measured at four southeast Australian forest OzFlux sites across three typical summer days (left) and three heatwave summer days (right) in 2019. Typical summer days were determined using historical summer climate data for southeast Australia, and the heatwave days were identified from Qiu et al. (2020). OzFlux sites include Tumbarumba (TUM, wet sclerophyll), Warra (WAR, wet sclerophyll), Whroo (WHR, dry sclerophyll) and Wombat State Forest (WOM, dry sclerophyll). Measurements are 30‐min ensemble averages from the four flux tower sites

Temperate and semi‐arid ecosystems in Australia display different mechanisms to tolerate prolonged water stress. For Mulga dominated semi‐arid ecosystems, extensive expression of ecophysiological adaptations allows survival through decadal scale droughts (Cleverly, Eamus, van Gorsel, et al., 2016; Eamus et al., 2013; Tarin et al., 2020b) and are usually reliant on single rainfall events to boost their CO_2_ uptake (Cleverly, Eamus, Restrepo Coupe, et al., 2016). Temperate ecosystems in non‐water limited regions of Australia are able to tolerate several years of below average rainfall through access to greater soil moisture reserves (Griebel, Bennett, et al., 2020; Keith et al., 2012; Kirschbaum et al., 2007). Access to soil moisture reserves helps buffer wet sclerophyll ecosystems against heatwaves, as illustrated by the combined drought and heatwave event in 2012/2013 that led to water‐limited woodland ecosystems becoming CO_2_ sources due to a reduction in photosynthesis caused by elevated water stress (Cleverly, Eamus, van Gorsel, et al., 2016; van Gorsel et al., 2016), while wetter forest systems were much less affected (van Gorsel et al., 2016). Model analysis of the more recent 2018/2019 heatwave showed reduced productivity for most ecosystems across continental Australia (Qiu et al., 2020). Four sites in southeast Australia also show reduced CO_2_ sink strength during this period (Figure 5). Some of these OzFlux observations are leading to much‐needed and rapid improvements in the CABLE LSM to better incorporate groundwater–vegetation interactions (Mu et al., 2021; Mu et al., 2021).

Drought can interact with disturbance (lesson 2) or other stress as was demonstrated at the temperate, wet sclerophyll, managed forest at Tumbarumba, where long‐term drought coincided with an insect attack (Kirschbaum et al., 2007). The forest was impacted by this attack, but it became a CO_2_ sink again when the insect attack had abated, despite continued and even intensifying drought conditions (van Gorsel et al., 2013). A future that consists of more frequent heatwaves in combination with drought could deplete soil moisture reserves beyond the tipping point for many ecosystems and result in greater ecosystem stress.

## 6 LESSON 4—ECOSYSTEM RESILIENCE, ADAPTATION AND VULNERABILITY TO INTERANNUAL CLIMATE VARIABILITY

Ecosystems can be resilient to climate variability by maintaining a stable carbon budget during and shortly following the imposition of stress (Holling, 1973) or through their capacity to rapidly recover to a pre‐stress state after the return of favourable environmental conditions (Ponce Campos et al., 2013). Because of Australia and New Zealand's contrasting climate zones and large interannual fluctuations in precipitation (Cleverly, Eamus, Luo, et al., 2016; Cleverly, Eamus, Restrepo Coupe, et al., 2016; Cleverly et al., 2019; Van Etten, 2009), measurements from across the OzFlux network are ideal to analyse and explore the effects of hydroclimatic variation (e.g. wet to dry seasons or years) on ecosystem carbon and water exchange (Karan et al., 2016). For example, while the strong interannual variability in arid and semi‐arid Australian ecosystems reduces productivity, its recovery does not appear to be limited by previous sequences of drought, swinging rapidly between states of net CO_2_ source and sink, sometimes from one year to the next (Cleverly, Eamus, Restrepo Coupe, et al., 2016; Cleverly, Eamus, van Gorsel, et al., 2016; Ma et al., 2016; Tarin, Nolan, Medlyn, et al., 2020). Due to the rapid recovery of Australian semi‐arid ecosystems following a year of extreme drought in 2009 (Cleverly et al., 2013; Cleverly, Eamus, Restrepo Coupe, et al., 2016; Eamus et al., 2013), these ecosystems contributed most to the observed global land carbon sink anomaly during the 2011 La Niña wet year (Poulter et al., 2014; Raupach et al., 2013).

Australian ecosystems also show resilience to drought and fire in their leaf phenology. For example, in Australia's mesic savannas, fire usually only consumes the seasonal grassy understorey, whereas canopy trees mostly remain intact (Lehmann et al., 2014). By contrast, in Australia's tropical drylands, a highly resilient leaf phenology allows strong growth during wet years despite the absence of a growing season in previous dry years (Ma et al., 2013). Similarly, Australian tropical rainforest trees are considered to be somewhat resilient to high‐temperature stress and heatwaves due to the very high temperature at which leaf dark respiration reaches a peak (60°C) (Weerasinghe et al., 2014), although they may be instead vulnerable to high VPD stresses (Fu et al., 2018). However, a loss of resilience has been predicted for Australian drylands with the increased occurrence of future woody dieback and megadrought events (Ma et al., 2013), and the continued resilience of many ecosystems in Australia and New Zealand is not assured with global change (van Gorsel et al., 2016).

Other examples of carbon‐function resilience to disturbance and drought are evident in managed and natural ecosystems of New Zealand. Here, dairy farm pastures have shown rapid recovery to a net positive carbon balance within one week following intensive grazing events. In these systems, grass is maintained in a continuously juvenile state through repeated grazing and defoliation by cattle (Hunt et al., 2016). In contrast, northern New Zealand's peat‐forming wetland ecosystems display resilience through the continuous accumulation of deep peat deposits over millennia, despite existing in a warm maritime climate zone with frequent seasonal water deficits. In the few remaining intact peat wetlands, resilience to drought is a product of the ecosystem's conservative evaporation regime and highly dynamic peat surface level (Campbell & Williamson, 1997; Fritz et al., 2008), both of which contribute to maintaining a stable and shallow water table, limiting respired CO_2_ losses (Goodrich et al., 2017; Ratcliffe et al., 2019). However, imposing artificial drainage diminishes their ability to self‐regulate, leading to a shift in ecosystem structure and function, resulting in larger component CO_2_ fluxes (Ratcliffe et al., 2019, 2020). Furthermore, resilience is completely lost when drained peatlands are used for dairy grazing, where annual CO_2_ losses can be extremely large, particularly during dry conditions (Campbell et al., 2015, 2021).

Despite these insights, there exist substantial gaps in our knowledge of the impacts of hydroclimatic variation on diverse natural and managed ecosystems that might yield clues about their resilience under the stresses imposed by changing climate. Some of these gaps result from the inadequate distribution of flux tower sites; for instance, the OzFlux network does not include sites within the indigenous native forests of New Zealand, and semi‐arid ecosystems are under‐represented in Australia (Beringer et al., 2016). Whilst research using OzFlux data has demonstrated the resilience of Australasian ecosystems to the large climate variability experienced in the past, much less is understood about their resilience to future global changes, especially larger and more frequent extreme weather events, warmer temperatures and changed rainfall regimes   that result from anthropogenic climate change.

## 7 LESSON 5—CLIMATE IMPACTS OF AGROECOSYSTEMS

Agriculture in New Zealand differs from many other countries in that since 1987, farmers have not been able to receive any government subsidies for production or environmental services associated with their ownership or stewardship of land. This forced farmers to rapidly become economically efficient and led to the growth of a commercially successful export‐oriented dairy industry (as well as other exporting agricultural and horticultural sectors). This dairy expansion, which has to a large extent replaced extensive sheep farming in the lower and flatter regions of the country, is overwhelmingly based on rotational grazing practice, involves active nutrient and feed supplement management and is in some drier regions supported with irrigation of pastures. Managing the land for food production has thus accelerated and intensified carbon, nutrient and water cycles and increased the country's agricultural greenhouse gas emissions by 17% from 1990 to 2019 (Ministry for the Environment, 2021).

The carbon budgets of agroecosystems are characterised by large exports of carbon in products such as grain, milk, meat or wool, as well as imports in fertilisers and animal excreta, in addition to the net ecosystem exchange (NEE) of carbon. To assess whether an agroecosystem gains or loses carbon over time, these exports and imports need to be quantified together with NEE to obtain the net ecosystem carbon balance (NECB). A productive system is usually a net CO_2_ sink, but there are examples from the OzFlux network where agroecosystems were a net carbon source (Laubach et al., 2019; Rutledge et al., 2017; Wall, Campbell, Morcom, et al., 2020; Wall, Campbell, Mudge, et al., 2020; Webb et al., 2018) due to net carbon exports exceeding NEE. These studies repeatedly suggest that the sign, strength and annual pattern of NECB are strongly impacted by farm management (Giltrap et al., 2020; Hunt et al., 2016; Rutledge et al., 2015; Wall, Campbell, Morcom, et al., 2020; Wall, Campbell, Mudge, et al., 2020). Agroecosystems on peat soils were both a net CO_2_ source and a net carbon source (Campbell et al., 2021; Goodrich et al., 2017).

Water fluxes are of critical concern in agroecosystems, where irrigation decisions are informed by balancing crop water use with yield‐based revenue, irrigation costs and regulatory limits for nutrient leaching. There are concerns that the practice of irrigation, increasingly widespread in NZ, may lead to net carbon losses, and soil‐core sampling studies point in this direction (Mudge et al., 2017). However, flux measurements over irrigated pasture did not find any carbon losses throughout the three years of measurements (Laubach & Hunt, 2018). In another study, capturing flux measurements over lucerne, it was found that total evaporation and drainage increased in response to irrigation, relative to a nearby non‐irrigated lucerne crop, with the benefit of larger biomass production at the cost of greater net carbon losses (Laubach et al., 2019). Recent modelling efforts calibrated with flux measurements have provided some insights into which combinations of livestock management and environmental factors lead to carbon gains or losses (Kirschbaum et al., 2017; Liáng et al., 2021). The degree and direction of coupling between evaporation and NEE can contribute to a greater understanding of agroecosystem function (Cleverly et al., 2020).

A globally significant consequence of agricultural food production is emissions of greenhouse gases, including CH_4_ (predominantly from ruminant animals and rice farming) and N_2_O (predominantly from microbial soil processes, stimulated by N addition with fertilisers and animal excreta). Technological challenges and instrumentation costs have limited the usage of the eddy covariance method for measuring fluxes of these non‐CO_2_ greenhouse gases; hence, other micrometeorological methods have predominantly been applied within the OzFlux network. Laubach and Hunt (2018) used a flux‐gradient technique to measure CH_4_ fluxes over three years at paired grazing sites in Canterbury, New Zealand, somewhat surprisingly finding that CH_4_ fluxes were consistently positive (i.e. the grazed pastures were a net source of methane) most of the time, even in the absence of cattle. Net emissions of similar magnitude have recently been found on farms in the north of NZ (J. P. Goodrich, pers. comm. 2021). The source of these CH_4_ emissions is unknown, and therefore it is not clear whether they are related to agricultural management. The flux gradient technique has also been applied to measure nitrous oxide emissions from dairy pasture (Laubach & Hunt, 2018). Wecking et al. (2020) compared N_2_O emissions, obtained with eddy covariance, to those calculated using locally determined emission factors, from small chamber plots treated with excreta and fertiliser. Both studies found that emission factors underestimated the N_2_O flux, since the chamber studies do not include N_2_O background emissions and possibly also due to a lack of seasonal variability in emissions factors (Laubach & Hunt, 2018; Wecking et al., 2020).

Studies of the fluxes from agroecosystems are gaining momentum as a robust approach to quantifying the efficacy of land management practices that aim to reduce or mitigate greenhouse gas emissions. To this end, paired sites approaches are promising (Laubach & Hunt, 2018; Laubach et al., 2019). Recent studies have overcome the cost of employing duplicate flux measurement systems with a split‐footprint approach (Goodrich et al., 2021; Wall, Campbell, Morcom, et al., 2020; Wall, Campbell, Mudge, et al., 2020), wherein an eddy covariance system is placed at the boundary between paired sites. Another possible approach lies in the development of low‐cost measurement systems (Hill et al., 2016). Communication between disciplines and with industry and policy makers will be central to OzFlux and the global flux community to help transition agricultural practices towards climate‐smart food systems.

## 8 LESSON 6—ADVANCES MADE VIA SYNERGIES WITH REMOTE SENSING

The initiation of OzFlux was shortly preceded by NASA’s Earth Observing System (EOS) that introduced the first suite of satellite‐based global ecology products for long‐term monitoring of ecosystem functioning, phenology, disturbance and plant stress (Xiao et al., 2019). The validity and robustness of these first biophysical products from remote sensing were challenged by the diversity of landscapes and extreme environments of Australia (Hill et al., 2006; Kanniah et al., 2009; Sea et al., 2011). For example, Leuning et al. (2005) reported that the moderate resolution imaging spectrometer (MODIS) LAI product overestimated in‐situ LAI more than twofold over the moderately open, wet sclerophyll forest at the Tumbarumba OzFlux site. These native forests are known for their highly clumped crown architecture and vertical leaf inclination angle (Anderson, 1981). The MODIS GPP product estimated the annual amplitude of tower GPP fluxes quite well but performed less well in estimating the seasonal phase of variation (Leuning et al., 2005). These assessments of remotely‐sensed products ultimately resulted in more accurate satellite products and understanding in what the satellite actually measures.

**FIGURE 6:**
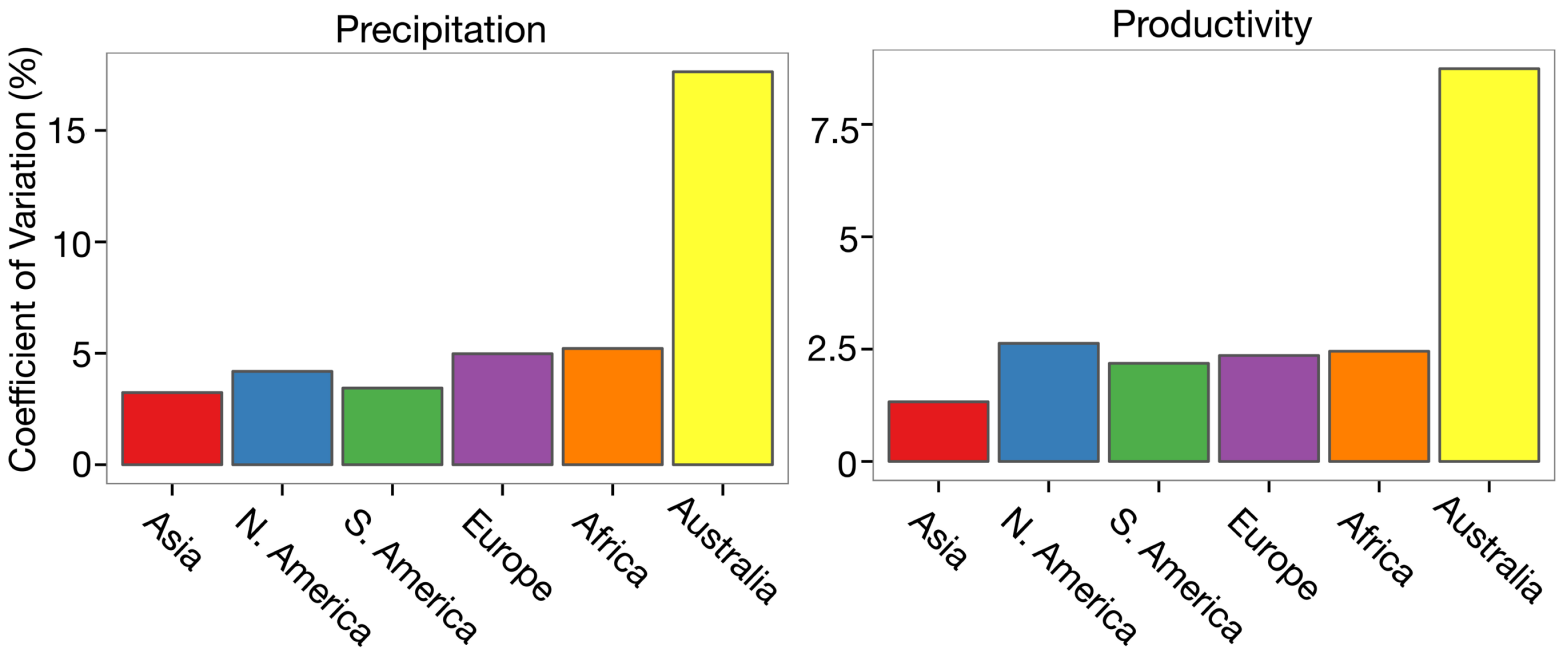
Coefficient of variation (%) in annual precipitation and annual vegetation productivity across six continents showing that Australia has a significantly higher variability in precipitation and corresponding productivity, as measured with the MODIS annual integrated EVI over a 15‐year reference period from 2000 to 2014. Reproduced with permission from Ma et al. (2016)

On the other hand, Sea et al. (2011) and Eamus et al. (2013) reported good agreement between MODIS LAI and hemispherical photography derived LAI in open‐canopied savanna ecosystems of the Northern Territory. MODIS vegetation indices (VIs) combined with meteorological data estimated GPP and latent heat flux (LE) with relatively high accuracy where ecosystem processes are phenologically driven, such as in Australian wet to dry tropical savannas, grasslands and croplands (Cleugh et al., 2007; Glenn et al., 2011; Ma et al., 2013; Moore et al., 2017; Zhang et al., 2008). However, in temperate and Mediterranean evergreen Australian forests/woodlands, the VI and LAI products were seasonally out of phase with GPP and found to be better proxies of photosynthetic ‘infrastructure’ capacity (Pc) than GPP (Restrepo‐Coupe et al., 2016). Broich et al. (2014) found extensive retrieval failures of the MODIS phenology product over the arid and semi‐arid regions of Australia, which led to the development of an Australian phenology product (https://portal.tern.org.au/) to better understand arid vegetation responses to Australia's climate extremes (Ma et al., 2015, 2016, 2016). Annually integrated VIs are a remote sensing surrogate of ecosystem productivity and have revealed the large sensitivity of interannual variations in productivity to precipitation variability in Australia, relative to all other continents (Figure 6; Ma et al., 2016).

Synergies between OzFlux and remote sensing have been used in diagnosing broad‐scale ecosystem responses to extreme events, including large scale, significant rainfall events that trigger continental‐scale green‐up of arid and semi‐arid ecosystems (see lesson 4). These continent‐wide green flushes can contribute significantly to the global land carbon sink and induce sea‐level anomalies, as occurred in 2010–2011 (Detmers et al., 2015; Fasullo et al., 2013). Such information is important in attributing the drivers of short term variability in the Earth system (e.g. are changes to the carbon sink due to human mitigation efforts or responses of the biosphere to prior events?). Ma et al. (2016) diagnosed this continental‐scale event by integrating multiple satellite measures of atmospheric CO_2_ (GOSAT), gravitational total water storage (GRACE), VIs (MODIS) and solar‐induced chlorophyll fluorescence (SIF, GOME‐2) with OzFlux tower derived NEP. They analysed the hydroclimate drivers and pulse response behaviour of carbon fluxes during the big wet and reported that semi‐arid Australian net CO_2_ uptake was highly transient and rapidly dissipated by subsequent drought. The accuracies of the remotely sensed CO_2_ retrievals and the atmospheric transport models are approaching the levels needed to constrain CO_2_ fluxes to estimate net biome productivity (NBP) from the natural biosphere (Buchwitz et al., 2017; Kondo et al., 2016).

The OzFlux network capitalises on skills and infrastructure through strong collaborations of people both at a national level and through international networks (Figure 2), including SpecNet (Gamon et al., 2006), https://specnet.info/tumbarumba/) and the Australian Phenocam Network (http://phenocam.org.au/). SpecNet sites are equipped with hyperspectral instruments and play important roles in linking in situ optical measures (fPAR, VIs and SIF) from tower platforms with flux observations, to explore mechanistic and scaling relationships (Leuning et al., 2006; Woodgate et al., 2020). The phenocam network enables high temporal image‐based recognition of understory/overstory dynamics at species levels, and thus enables leaf level demography, ontogeny and phenology analyses (Moore, Brown, et al., 2016; Wu et al., 2016). These sub‐daily, near‐ground spectral and phenocam measurements bridge temporal, spatial and spectral scales with airborne and satellite remotely sensed proxies of canopy and ecosystem function.

Capturing the range of global variability in ecosystems is critical for accurately calibrating, validating and upscaling satellite algorithms and modelled outputs using high‐quality ground‐level data. In a global flux tower analysis using MODIS satellite products and meteorological drivers, Tramontana et al. (2016) found that carbon and water fluxes from extreme climates and Southern Hemisphere flux sites were less accurately simulated than Northern Hemisphere forested and temperate climate sites. The OzFlux sites, located in globally under‐represented areas, have the potential to reduce these uncertainties in global carbon and water flux products. OzFlux sites account for a large proportion of global land surface FLUXNET observations in biomes located at high mean annual temperatures and with extreme climate variability, as shown in Figures 1 and 3) (Van Der Horst et al., 2019), making them crucial for the validation of new satellite sensors, novel algorithms and in the development of national and global products and models (Barraza Bernadas et al., 2018; Barraza et al., 2015, 2017; Guerschman et al., 2009; Pham et al., 2019; Sanders et al., 2016; Verma et al., 2017; Zhang et al., 2019).

While early remote sensing work was focussed primarily on VIs and LAI, an increasing number and diversity of observations can now target specific components of the terrestrial carbon cycle and water cycle at high temporal and spatial resolution (Schimel & Schneider, 2019). For example, the use of SIF and VIs together can be used to disentangle controls of canopy structure from physiology on GPP (Magney et al., 2019; Springer et al., 2017; Verma et al., 2017). This is particularly important for evergreen canopies (dominant in Australia and New Zealand) where GPP is often decoupled from VIs (Restrepo‐Coupe et al., 2016).

The current generation geostationary satellites (e.g. Himawari‐8) provide sub‐daily, 10‐min image acquisition frequencies in near real‐time across Australia, enabling integration with diurnal fluxes for refined insights into ecosystem dynamics. A metric of canopy structure, canopy clumping index, was recently retrieved from sub daily measures from the Deep Space Climate Observatory (DSCOVR) satellite and evaluated at OzFlux sites (Pisek et al., 2021). The International Space Station (ISS) has three instruments that provide regional‐ diurnal measures of (1) Evapotranspiration from the ECOsystem Spaceborne Thermal Radiometer Experiment (ECOSTRESS) at 70 m resolution; (2) SIF from the Orbiting Carbon Observatory‐3 (OCO‐3), at 100 m resolution and (3) Biomass and canopy structure from the Ecosystem LiDAR Global Ecosystem Dynamics Investigation (GEDI) instrument, at 25 m to 1 km resolutions (Xiao et al., 2021). Together these instruments provide unprecedented opportunities to assess diurnal variations in GPP, ET (evapotranspiration, the mass equivalent of LE) and thereby water use efficiency (WUE) at different times of day, with OzFlux sites being critical to validate these products (Fisher et al., 2020; Xiao et al., 2021). Other sensors include soil moisture mapping, vegetation optical depth, atmospheric trace gases (CO_2_, CH_4_, CO) for inversion studies and advanced hyperspectral sensors for canopy traits. These new remote sensing advances will be vital to scale knowledge (Figure 2) of ecosystem processes from OzFlux sites to landscape and continental scales in the context of climate change.

## 9 LESSON 7—ADVANCES MADE VIA SYNERGIES WITH MODELLING

One of the most important outcomes from OzFlux has been the ability to constrain models used to quantify and predict terrestrial carbon and water fluxes, from site‐scales (Kirschbaum et al., 2007, 2015) to the continent (Decker, 2015), using multi‐annual, continuous data from around Australia and sampling a range of bioclimates. Foremost among these outcomes was the construction of a full continental carbon budget for Australia (Haverd, Raupach, Briggs, Canadell, Davis, et al., 2013). This work used multiple data sources, including OzFlux data, to constrain the CABLE LSM (Wang et al., 2011). The data‐constrained estimate of Australia's NBP for 1990–2011 was 36 ± 29 Tg C yr^−1^ (Haverd, Raupach, Briggs, Canadell, Davis, 2013; Haverd, Raupach, Briggs, Canadell, Isaac, et al., 2013), with annual net primary productivity (NPP) quantified at 2.2 ± 0.4 Pg C yr^−1^.

Similarly, OzFlux data underpin operational water modelling in Australia. Although potential evaporation can be quantified from a spatial network of pan evaporation data dating back to 1975 (Roderick & Farquhar, 2004; Stephens et al., 2018), OzFlux sites provide the only observations of actual evapotranspiration (AET). OzFlux AET data were used in the evaluation of modelled evapotranspiration in the operational AWRA model used for the Australian Bureau of Meteorology's water information services (van Dijk, 2010; Frost et al., 2015). OzFlux data have also been used to constrain large‐scale AET estimates from process‐ and satellite‐based models, yielding a data‐constrained estimate of mean Australian AET over the period 2000–2010 of 360 ± 205 mm yr^−1^ (Hobeichi et al., 2021). The marked uncertainty in continental‐scale estimates of Australia's terrestrial carbon and water fluxes not only stems in part from the inherent climate variability (lesson 1) but also underlines the challenges faced in advancing our understanding of Australia's terrestrial biogeochemical cycles and budgets.

OzFlux data have also been an important resource to benchmark, evaluate and improve model formulations at time scales ranging from sub‐daily (Abramowitz, 2012; Haughton et al., 2016) to interannual (Wang et al., 2011). The coverage of extreme events in the dataset has been of significant value (De Kauwe et al., 2019; Yang et al., 2019). The high interannual variability in rainfall has enabled the use of OzFlux data to uncover systematic biases in LSMs in simulating carbon and water fluxes during drought (Haverd, Ahlström, et al., 2016; Haverd, Smith, Raupach, et al., 2016; Haverd, Smith, Trudinger, et al., 2016; Li et al., 2012; Torre et al., 2019; Ukkola et al., 2016), identifying priorities for model development to reduce uncertainties in future projections of drought (De Kauwe et al., 2020; Stocker et al., 2018) and water resources.

The unique coverage of the savanna biome provided by the North Australian Tropical Transect component of OzFlux has helped identify limitations in terrestrial biosphere models in representing savanna ecosystems (Haverd, Smith, Raupach, et al., 2016; Whitley et al., 2016), providing directions for improving the modelling of savannas globally (Whitley et al., 2017). The phenology of leaf area, root water uptake and disturbance from fire were highlighted as key areas of uncertainty for future research.

The open‐access availability of OzFlux data has enabled immediate improvements to a diversity of models. For example, AET data were used to reformulate the representation of soil evaporation during the wet season, resulting in significant improvements in AET predictions of the GRASP suite of models used operationally in Queensland for pasture and grazed woodland systems (Owens et al., 2019). However, OzFlux data have principally been used to evaluate models, rather than to drive theory development. This gap exists because ancillary site measurements needed to interpret the measured fluxes in the right (ecosystem‐specific) context are often lacking (e.g. plant physiological and structural traits, phenology, biomass, LAI and soil moisture). To address this shortcoming, future focus should lie on the provision of a standardised set of these ancillary measurements at regular time intervals. The founding of Australia's first Critical Zone Observatory—a monitoring network covering the top of the tree canopy to the groundwater—at five sites across Australia—aims to make a significant contribution towards reducing scaling uncertainties over the next decade (De Kauwe et al., 2017; Medlyn et al., 2017).

There are several obvious opportunities to develop new model theory. Linking OzFlux data, particularly sites with concurrent measurements of (deep) soil moisture (e.g. the wet sclerophyll forest site, Wombat, in southeast Australia) with satellite remote sensing, would enable the development of new theory to understand leaf growth dynamics under changing water availability. Measurements of hydraulic traits across the OzFlux network (Peters et al., 2021), coupled with eddy covariance data, would facilitate the development and testing of new theories governing plant controls on transpiration. A key question relates to how the carbon and water cycles will change in the future; answering this will require longevity across the OzFlux and the wider FLUXNET network.

## 10 LESSON 8—THE IMPORTANCE OF LONG‐TERM MEASUREMENTS TO DETECT DECADAL SCALE EVENTS AND CLIMATE CHANGE EFFECTS

Given the geographical extent of the Australian and New Zealand regions and the associated large range of climate drivers, climatic variability is naturally high (King et al., 2020), and this variability is increasing due to changes in climate and land use (Head et al., 2014; King et al., 2020). Regional climate variability is also driven by complex, large‐scale ocean–atmosphere influences that operate at frequencies from weeks to decades and have a strong influence on rainfall (King et al., 2020; Rogers et al., 2017), and therefore drives variability of ecosystem dynamics (Cleverly, Eamus, Luo, et al., 2016) (See also lesson 2). The net result is a climate system which operates in widely varying states spatially and temporally, driving periods of drought, flood and heatwaves (Freund et al., 2017; Kiem et al., 2016; Perkins‐Kirkpatrick et al., 2016) that are increasing in severity with climate change (Cai et al., 2014, 2021). Extreme events have a disproportionate effect on annual carbon exchange at regional to continental scales (Zscheischler et al., 2017) and long‐term monitoring of ecosystem carbon exchange, water use and resource use efficiency is required to understand and predict ecosystem responses to the changing climatic range. This is particularly important in Australia, which is a global hot spot for variability—especially in semi‐arid ecosystems, which exhibit large and ‘asymmetrical’ responses of GPP to rainfall variability (Haverd, Ahlström, et al., 2016). This large interannual variability makes detecting long‐term trends from short records extremely difficult (Baldocchi et al., 2018). On the other hand, Australia may also provide an example to inform other continents about how ecosystems will adapt to increased climate variability with resource availability hard to predict.

**FIGURE 7:**
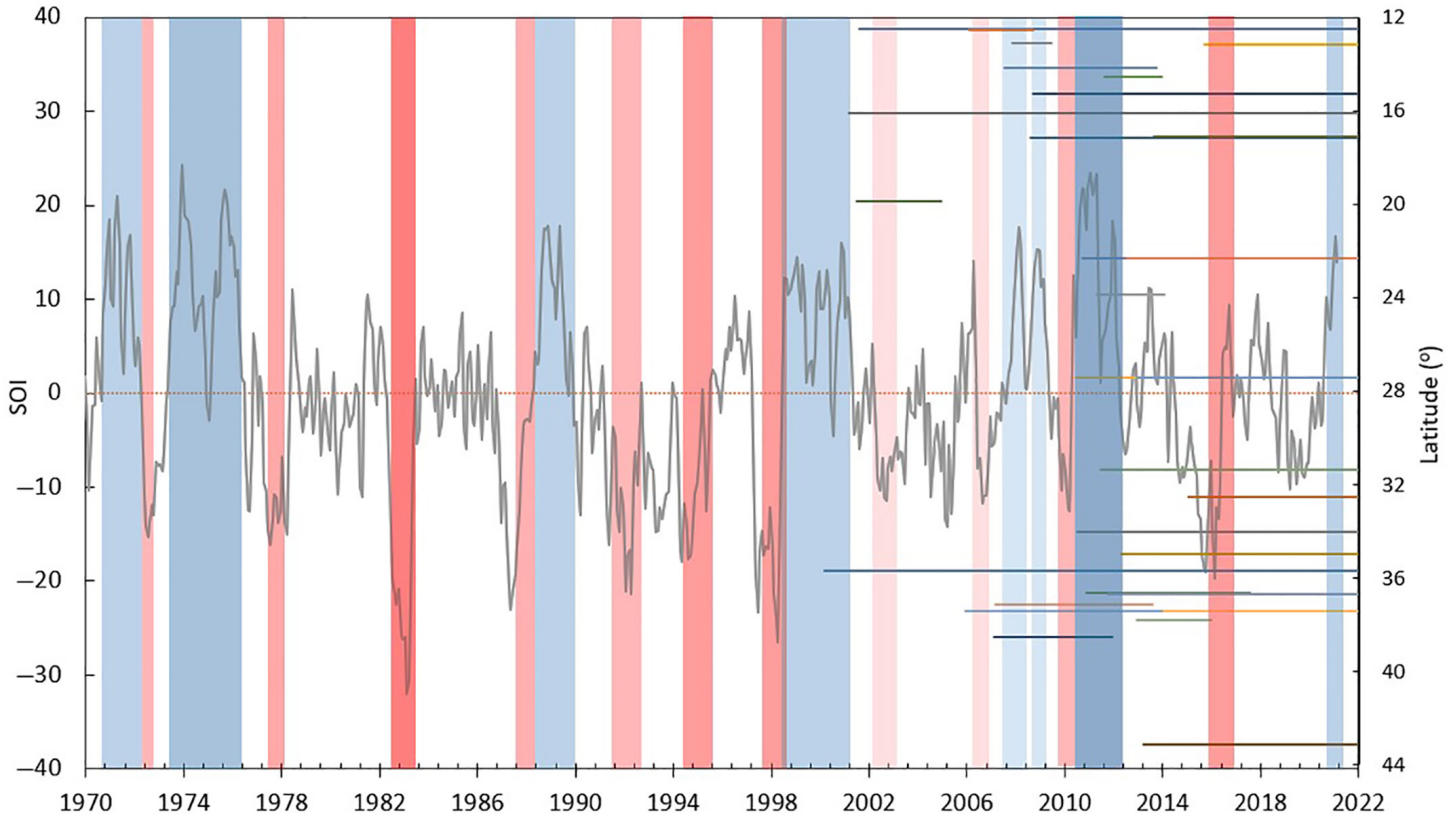
Monthly SOI record from 1970 to 2021 with key El Niño (red bars) and La Niña (blue bars) events that led to severe flooding, drought and fire events in Australia. Bar colours represent event severity (strong, moderate or weak). Overlaying the SOI timeseries is the observation periods for all previous and current Australian OzFlux and Supersites plotted as coloured lines using site latitude. Site data durations were taken from the TERN OzFlux data portal (www.ozflux.org.au/monitoringsites/index.html) and ENSO periods were taken from the Australian Bureau of Meteorology (www.bom.gov.au/climate/enso/enlist)

A comprehensive understanding of interannual and interdecadal variability of the carbon cycle and its drivers requires long‐term data (>50 years) (Fu et al., 2019; He et al., 2019; Jung et al., 2017; von Buttlar et al., 2018; Zscheischler et al., 2016). Continued operation of existing sites and the expansion of the global eddy covariance monitoring network (Baldocchi, 2019), together with the increasing length of the satellite record, will provide the observational constraints to gain this understanding. The two decades of observations in the OzFlux network span several significant ENSO events (Figure 7), and this length of record can be used to detect change in ecosystem properties as a function of short‐term or high‐frequency disturbances such as fire, insect attack, drought and cyclones (Beringer et al., 2007; Hutley et al., 2013; Keith et al., 2012). The network has captured fluxes during the ‘Millennial Drought’ from 1997 to 2009 that was followed by the globally significant southern hemisphere La Nina of 2010/2011, the severe El Niño event of 2015/2016, the unusually hot and dry spring of 2019, and flooding associated with the 2021 wet season across the southeast Australian seaboard. However, in terms of long‐term climate trends, OzFlux has only a few sites with 20 years of data.

**FIGURE 8:**
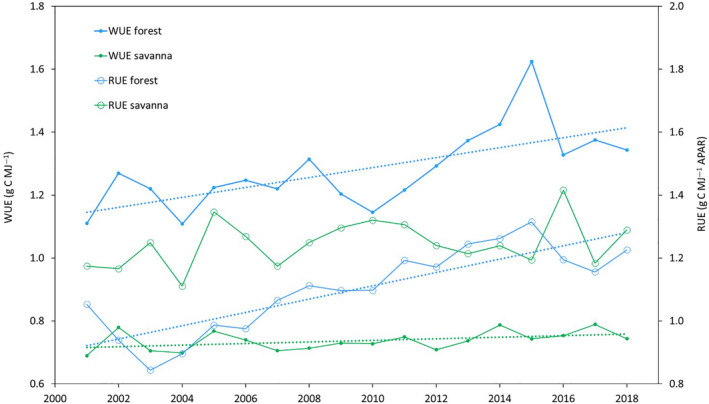
Timeseries of observed ecosystem water use and radiation use efficiency from two OzFlux sites with 20‐year records: tropical savanna at the Howard Springs site and temperate Eucalypt forest at the Tumbarumba site. Trend lines are given for significant time series (*p* < 0.05) using the non‐parametric Mann Kendal test

The responses and interannual variability of two long‐term but contrasting OzFlux sites is shown in Figure 8, where we illustrate trends in water‐ and radiation‐use efficiencies (WUE=GPP/LE, RUE=GPP/APAR) for a managed, temperate mixed Eucalypt forest (AU‐Tum) and a tropical savanna in the NT (AU‐How). To estimate absorbed PAR (APAR) for each site, we used the MODIS 8‐day, 500 m resolution fractional absorbed photosynthetically active radiation product (fPAR, MOD15A2) interpolated to provide a daily estimate of fPAR which was then used to scale daily measures of short‐wave radiation after Garbulsky et al. (2010).

WUE is ~30% higher in the temperate, wet sclerophyll forest at Tumbarumba (AU‐Tum) than the tropical savanna at AU‐How, which is surprising given C4 grasses (high WUE) dominate the understory of the savanna ecosystem. However, these grasses are largely annual and are only active 4–5 months of the wet season, whereas the evergreen C3 woody species of Australia's temperate forests are active all year (Eamus et al., 2001; Moore, Beringer, et al., 2016). Frequent savanna fires (2 in 3 years) scorch the woody canopy and post‐fire canopy reconstruction results in high respiratory losses (Cernusak et al., 2006) with the ecosystem a net source of CO_2_ for months after fire, whereas LE recovers within weeks (Beringer et al., 2007). This post‐fire recovery phase is a period of lower WUE, and the savanna ecosystem has a lower‐than‐expected WUE because of these ecosystem characteristics.

Trends in WUE and RUE are highly statistically significant at AU‐Tum (*p *< 0.01), and WUE increased by 16% over 18 years, whereas the tropical savanna site only increased by 6% (Figure 8). Over the period of observation, atmospheric CO_2_ concentrations increased by about 10%, and the trend in WUE at AU‐How is consistent with theoretical expectations of increased photosynthesis and WUE (Kirschbaum & McMillan, 2018; Walker et al., 2021). However, the trend at AU‐Tum (16% for WUE, 30% for RUE) exceeds what could be reasonably attributed to CO_2_ fertilisation alone, suggesting recovery from disturbance events (e.g. insect outbreaks, van Gorsel et al., 2013) plus increasing efficiency as the stand ages and grows in response to commercial forestry activities.

The spatial and temporal limitations of the OzFlux network highlight the importance of integrating long‐term flux observations with remote sensing and modelling studies (lessons 6 and 7). As climate variability increases, there is a clear imperative to maintain long‐term monitoring sites and invest in modelling systems structured to the physiological properties of Australian and New Zealand vegetation to assess their response to increasing climatic variability and disturbance. Australian ecosystems have shown a degree of resilience to date (De Kauwe et al., 2020), but only long‐term data will enable us to detect tipping points across the spectrum of Australian and New Zealand ecosystems and improve our ability to forecast potential systematic ecological changes (Bergstrom et al., 2021; Laurance et al., 2011). Assessing cumulative long‐term impacts on diverse ecosystems is critical for the management of both natural and food production systems. It is, therefore, crucial to maintain the existing network to ensure the continuity of flux data and increase the number of long‐term sites into the future.

## 11 THE STRENGTH OF OzFlux AND OUR VISION FOR THE FUTURE

The IPCC’s Sixth Assessment Working Group I Report (IPCC, 2021) documents an increased rate and greater certainty of global warming relative to previous assessments. Australia's climate has already warmed by 1.44°C since national records began in 1910 (Australian Bureau of Meteorology & CSIRO, 2020) and although we have shown that Australian ecosystems currently have some resilience, the increased frequency and intensity of climate extremes, and an emerging drying trend in the southern part of the continent, have the potential to push some ecosystems (e.g. temperate forests) over tipping points (Perkins‐Kirkpatrick et al., 2016). As such there is a growing imperative to use and build on our knowledge of ecosystem processes and emergent phenomena (Karan et al., 2016). These processes must be studied across a range of temporal and spatial scales to be properly understood and integrated into modelling. Synergistic network science has allowed these emergent processes to be understood, as patterns in space and time are revealed by multiplying manifold observations across numerous individual researchers and sites.

The need to continue operating OzFlux and other ecosystem observatories is increasingly important to (1) inform the science and models needed for accurate ecological forecasts and longer‐term projections of responses to climate extremes; (2) document recovery from disturbances, and evaluate potential new land management strategies and longer‐term trends in the effects of observed climate change and variability—this demands multi‐decadal and continuous observations; (3) diagnose interannual variability in the carbon cycle and net greenhouse gas emissions, and verify carbon market products and greenhouse‐gas mitigation approaches; (4) evaluate and improve models of terrestrial ecosystem feedbacks to climate change, and (5) evaluate and improve simulations of the feedbacks between the land and atmosphere in the context of short‐duration heatwaves and drought.

Ecosystems are expected to experience continued long‐term climate change and greater variability along with increased disturbance leading to a loss of ecosystem services. To best maintain our ecosystems and their services, we must anticipate and plan for these changes using predictive modelling and ecological forecasting. Developing this capability is crucial and will require forecasting (over the near term) and projections over multidecadal time scales) using real‐time flux information (OzFlux), ecological observing infrastructure (e.g. TERN), new and emerging satellite information and a new iterative model forecasting paradigm (Dietze et al., 2018). Australia's 2016 National Research Infrastructure Roadmap also identified a need to establish a National Environmental Prediction System (https://science.uq.edu.au/neps). This could facilitate integration of environmental observations with predictive modelling, thus improving environmental risk management. New streams of earth observing satellite data are emerging from advanced sensors. However, the interpretation of their underlying ecological signals requires continued validation with ground‐based sensors and leaf‐level measurements. Using spectral indices and more direct observations of vegetation productivity through SIF provide excellent prospects for better detection of ecosystem stress (e.g. NASA ECOSTRESS, ESA FLEX). OzFlux will continue to participate as a key provider of ground stations in the Southern Hemisphere and will provide opportunities for further synergies between remote sensing and ecosystem ecologists.

Ongoing collaboration between ecophysiologists and ecosystem flux researchers is leading to improved mechanistic understanding of the role of the terrestrial vegetation in the annual and inter‐decadal hydrologic cycle and the carbon balance across a wide range of ecosystems.

Of emerging interest is the connection of physiological/hydraulic traits to the dynamic role of the subsurface in regulating surface ecosystem fluxes and vegetation health. For example, an increasing body of international evidence illustrates how groundwater, deep soil moisture (Mu, De Kauwe, Ukkola, Pitman, Guo, et al., 2021, Mu, De Kauwe, Ukkola, Pitman, Gimeno, et al., 2021) and rock moisture (Hahm et al., 2019; McCormick et al., 2021) constrain the interannual variability of plant water use and productivity, potentially buffering ecosystems from water stress imposed by climate change (McLaughlin et al., 2017). Similarly, plant hydraulic models are revealing how the interaction of plant physiological traits with climate and soil at a given site, rather than these factors in isolation which control the risk of drought mortality (Feng et al., 2018, 2019). In the future, measurements of hydraulic traits across the OzFlux network (Peters et al., 2021), coupled with eddy covariance data, could facilitate the development and testing of new theories governing plant controls on transpiration.

A significant proportion of Australia's total ecosystem biomass (ca. 30%–50%, Spawn et al., 2020) is found in the subsurface, yet our understanding of how the subsurface environment changes and influences ecosystems is lagging. Newly funded critical zone observatories (CZOs), co‐located at several OzFlux sites, are now installing the equipment to monitor water, carbon and energy throughout deep soil profiles. By integrating observations of subsurface variation with the surface fluxes measured by OzFlux, these CZOs will offer better understanding of the interdependencies of carbon and water cycles across timescales and across the full vertical span of Australian ecosystems.

Ecosystem observatories are moving beyond CO_2_ and water cycles to monitoring other greenhouse gases, especially emissions of CH_4_ from wetlands and N_2_O from agricultural systems as highlighted in the lessons above. These potent greenhouse gases can now be measured at temporal and spatial scales that are relevant to land management and planning for mitigation of climate change.

There is currently a high demand for new researchers with skills in environmental monitoring, sensors and data analysis; however, it is a challenge to sustain training of postgraduate students and our capacity in the discipline of global change biology. Recruitment of new talent needs to start at the undergraduate level or earlier, to ensure a flow of quantitatively skilled researchers who are passionate about ecosystem science. Educational collaborations among engineers, atmospheric scientists, hydrologists, ecologists, physicists and others will set the stage for the next generation of environmental leadership and stewardship. OzFlux will continue to play a major role in training this next generation and in providing the ecosystem data which scientists, the public and managers/government can rely on in understanding our rapidly changing environment in Australia and New Zealand.

## AUTHOR CONTRIBUTIONS

This manuscript was designed after a 20 years of OzFlux celebration conference held in 2020. The manuscript was prepared by Beringer, Moore and Cleugh with input from all co‐authors. Each lesson was compiled by a section lead as follows; Cleugh led ‘the genesis of OzFlux’, Griebel led Lesson 1, Cleverly led Lesson 2, Moore led Lesson 3, Campbell led Lesson 4, Grover and Laubach led Lesson 5, Huete and van Niel led Lesson 6, De Kauwe and Kirschbaum led Lesson 7 and Hutley led Lesson 8.

## Data Availability

The OzFlux data that support the findings of this study are openly available from the OzFlux data portal at https://www.ozflux.org.au/. Specific sites used were Tumbarumba (https://doi.org/102.100.100/14241), warra (https://doi.org/102.100.100/22566), whroo (https://doi.org/102.100.100/14232), Wombat State Forest (https://doi.org/102.100.100/14237), and Howard Springs (https://doi.org/102.100.100/14234). Data to support production of Figure 5 were also provided by the Australian Bureau of Meteorology via www.bom.gov.au/climate/enso/enlist.
